# *Candida dubliniensis* endophthalmitis: five cases over 15 years

**DOI:** 10.1186/1869-5760-3-66

**Published:** 2013-11-19

**Authors:** Thomas P Moloney, Joseph Park

**Affiliations:** 1 Department of Ophthalmology, The Royal Brisbane and Women's Hospital, Herston, Brisbane, Queensland 4006, Australia

**Keywords:** Endophthalmitis, Fungal endophthalmitis, *Candida*, *Candida dubliniensis*

## Abstract

**Background:**

Recent studies have shown that the recently identified organism *Candida dubliniensis* is less pathogenic than the more common *Candida albicans*. Due to its rare nature, *C. dubliniensis* has been previously reported as the causative organism in endophthalmitis in only three cases. We undertook a multicenter, retrospective, consecutive case series to describe the clinical features and outcomes of patients with culture-proven *C. dubliniensis* endophthalmitis. Medical records were reviewed for all patients with *C. dubliniensis* endophthalmitis on vitreous/aqueous cultures from June 1998 to June 2013 from all public hospitals throughout Queensland, Australia.

**Results:**

Six eyes from five patients were identified - four males and one female aged from 21 to 55 years (mean 37 years). Four patients were intravenous drug users and four patients had hepatitis C. All five patients were treated with systemic antifungal therapy and intravitreal antifungal injections, and all required vitrectomy. Two eyes developed retinal detachment over the course of the endophthalmitis. Five eyes had visual outcomes of 20/60 or better, and one eye had a poor outcome with final visual acuity of hand movements only. There was no associated mortality, and no infected eyes required enucleation or evisceration.

**Conclusions:**

*C. dubliniensis* endophthalmitis is a rare condition which occurs mainly in intravenous drug users and can occur in both HIV-positive and HIV-negative patients. Unlike *C. albicans* endophthalmitis, *C. dubliniensis* endophthalmitis has reasonable visual outcomes and does not appear to be associated with high mortality.

## Background

Endogenous fungal endophthalmitis is a rare, potentially blinding complication of systemic fungal infection. Overall, the commensal yeast *Candida albicans* is the most common fungal isolate in patients with endogenous fungal endophthalmitis, although other *Candida* strains have been implicated as causative organisms including *Candida tropicalis*, *Candida parapsilosis*, *Candida glabrata*, *Candida guilliermondii*, *Candida krusei*, and more recently *Candida dubliniensis*[1]*. C. dubliniensis* was first identified in 1995 in Ireland as an oral commensal isolated from HIV-infected individuals and has since been isolated in a variety of other candidal infections [2]. Although *C. dubliniensis* was initially identified as an ‘atypical’ form of the more common *C. albicans*, subsequent phenotypic and genotypic studies have identified it as a separate strain [2]. Further studies have shown that *C. dubliniensis* and *C. albicans* differ not only phenotypically but also in terms of epidemiology, virulence characteristics, and the ability of *C. dubliniensis* to develop fluconazole resistance [3]. Due to these variations, it is important to differentiate these strains in clinical situations like *Candida* endophthalmitis due to potential differences in presentation, treatment, and clinical outcomes. To our knowledge, there have been only three previously reported cases of endophthalmitis caused by *C. dubliniensis* since the discovery of the organism [4-6]. We report and discuss the significance of five new cases of *C. dubliniensis* endophthalmitis identified over 15 years in public hospitals in Queensland, Australia.

## Methods

Queensland public hospital pathology records were reviewed for all patients with endophthalmitis and vitreous/aqueous samples that cultured *C. dubliniensis* over a 15-year period from June 1998 to June 2013. Five patients were identified over this period. The medical records of these patients were then retrospectively reviewed for demographic data, background medical history, presenting signs and symptoms, diagnostic testing, microbiology results, treatment received, visual outcome, and mortality. This review was conducted in accordance with guidelines set forth by the Declaration of Helsinki and was exempt from institutional review board approval.

## Results

### Age, sex, and predisposing factors

Six eyes from four males and one female aged from 21 to 55 years (mean 37 years) were identified for our series (Tables 1 and 2). All patients had endogenous endophthalmitis, but only two patients had other symptoms of systemic illness with the other three patients having isolated endophthalmitis only. Four patients had a background of intravenous drug use (80%), four patients had hepatitis C (80%), one patient had associated liver cirrhosis (20%), and one patient had associated *Candida* endocarditis (20%). The four patients with hepatitis C were not being treated with antiviral therapy at the time of presentation. Two patients had intravenous lines *in situ* at the time of presentation (40%). All five patients had no previous ophthalmic history, and all had best-corrected visual acuities of better than 20/32 in both eyes before presentation for endophthalmitis.

**Table 1:** **Endogenous *C. dubliniensis *** **endophthalmitis cases**

**Case (reference)**	**Sex, age**	**Comorbidities**	**Site**	**Referral pathway**	**Initial visual acuity**	**Antifungal sensitivity**	**Antifungal treatment and surgery**	**Final visual acuity**
Sedeek, 2008 [4]	M, 38	Nil	Right eye	Not reported	VA RE - HM, LE - 20/20	Fluconazole, voriconazole, caspofungin, amphotericin B	Urgent lensectomy/vitrectomy, IVI vancomycin/ceftazidime. IVI/topical amphotericin B, PO voriconazole (no duration)	Not reported
Pelegrin, 2010 [5]	M, 41	IVDU, HIV+, hepatitis B and C, fever, neutropenia	Right eye	Presented to eye emergency	VA RE - 20/200, LE - 20/400	Azoles	Vitrectomy, IVI amphotericin B. Systemic voriconazole then PO fluconazole for 2 months	VA RE - 20/60
Espinosa-Heidmann, 2012 [6]	M, 27	IVDU, onychomycosis	Left eye	Presented to eye clinic	VA LE - 20/400	Fluconazole and all other agents tested	Toxoplasma treatment. IV fluconazole. Vitrectomy/IVI amphotericin B. PO fluconazole for 6 weeks	VA LE - 20/80
Present case 1	M, 50	IVDU, hepatitis C	Right eye	Walk-in to eye outpatients	VA RE - CF, LE - 20/20	Fluconazole, 5-flucytosine, voriconazole, amphotericin B	Empirical IVI amphotericin B. Vitrectomy. 2× IVI amphotericin B. PO voriconazole for 46 days	VA RE - 20/30, LE - 20/16
Present case 2	M, 28	IVDU	Right eye	Walk-in to eye outpatients	VA RE - 20/200, LE - 20/20	Fluconazole, 5-flucytosine, voriconazole	Urgent vitrectomy/IVI voriconazole. Empirical systemic voriconazole. 3× IVI amphotericin B. Vitrectomy/buckle/gas. PO fluconazole for 42 days	VA RE - 20/60, LE - 20/20
Present case 3	M, 34	IVDU, hepatitis C, endocarditis, PICC	Right eye	Inpatient referral	VA RE - HM, LE - 20/60	Fluconazole, 5-flucytosine, voriconazole	Empirical fluconazole, IVI amphotericin B. 2× IVI amphotericin B. Vitrectomy. PO voriconazole for 35 days. Vitrectomy/buckle/gas	VA RE - HM, LE - 20/20
Present case 4	M, 55	T2DM, hepatitis C, liver cirrhosis, PICC	Bilateral	Inpatient referral	VA RE - 20/200, LE - 20/200	Fluconazole, 5-flucytosine, voriconazole, amphotericin B	Empirical fluconazole. Right vitrectomy. 10× IVI voriconazole RE. 4× IVI voriconazole LE. Left vitrectomy. PO fluconazole for 42 days	VA RE - 20/60, LE - 20/60
Present case 5	F, 21	IVDU, hepatitis C	Left eye	Walk-in to eye outpatients	VA RE - 20/18, LE - 20/200	Fluconazole, 5-flucytosine, voriconazole, amphotericin B	Empirical voriconazole, IVI amphotericin B. 8× IVI voriconazole. Vitrectomy. PO fluconazole for 36 days	VA RE - 20/16, LE - 20/30

*Abbreviations:CF* counting fingers, *F* female, *HM* hand movements, *IVDU* intravenous drug use, *IVI* intravitreal injection, *LE* left eye, *M* male, *PICC* peripherally inserted central catheter, *PO* per oral, *RE* right eye, *T2DM* type 2 diabetes mellitus, *VA* visual acuity.

**Table 2:** **Summary statistics for endogenous *C. dubliniensis *** **endophthalmitis**

	**Queensland series**	**Previous endogenous cases**	**Total**
Number of patients	5	3	8
Number of eyes involved	6	3	9
Diagnosis and microbiology			
Vitreous taps	5	1	6
Positive fungal vitreous taps	3 (60%)	1 (100%)	4 (67%)
Vitrectomy specimens	7	3	10
Positive fungal vitrectomy specimens	3 (43%)	3 (100%)	6 (60%)
Ocular treatment			
Intravitreal amphotericin (number of eyes)	3 (50%)	2 (66%)	5 (56%)
Intravitreal voriconazole (number of eyes)	4 (66%)	0	4 (44%)
Urgent vitrectomy	1 (17%)	1 (33%)	2 (22%)
Total number of vitrectomies	8	3	11
Systemic treatment			
Empirical fluconazole	2 (40%)	1 (33%)	3 (38%)
Empirical voriconazole	2 (40%)	1 (33%)	3 (38%)
Definitive fluconazole therapy	3 (60%)	2 (66%)	5 (56%)
Definitive voriconazole therapy	2 (40%)	1 (33%)	3 (33%)
Mean duration of antifungal treatment (days)	40	49	43
Outcomes			
Mean duration of follow-up (days)	345	56	287
Final best-corrected visual acuity better than 20/80	5 (83%)	2 (100%)	7 (87%)^a^
Final best-corrected visual acuity worse than 20/200	1 (17%)	0	1 (13%)^a^
Retinal detachment	2 (33%)	0	2 (22%)
Enucleation/evisceration	0	0	0
Mortality	0	0	0

^a^Details of final visual acuity have been described only in eight cases.

### Presentation and diagnosis

In terms of referral pathway, three patients presented to the hospital ophthalmology outpatient department with a mean time from onset of symptoms to ophthalmologic review of 6.3 days. Two patients were current hospital inpatients, and the mean time from onset of symptoms to ophthalmologic review in these patients was 1 day. The right eye was affected in three cases and the left eye in one case, and there was one case of bilateral endophthalmitis. Visual acuity was 20/200 or worse in the affected eye in all patients at presentation (Table 1). The major presenting symptom in all cases was decreased visual acuity. On examination, all patients had severe anterior chamber inflammation and severe vitritis. No patient presented with hypopyon. Two patients had evidence of vitreous snowballs.

### Diagnostic testing and microbiology

Systemically, all patients had blood cultures, HIV serology, and echocardiography on presentation. A total of 19 blood cultures were collected from the five patients (mean 3.8 per patient, range 1–8). Only 4 of these 19 cultures were positive for *C. dubliniensis* (22%), and all 4 of these were collected from the patient with bilateral endophthalmitis. All patients were HIV negative on serology at the time of presentation. Echocardiography showed associated endocarditis in one patient.

In terms of ophthalmic investigations, between the six eyes, five vitreous taps were performed with three samples (60%) producing positive *C. dubliniensis* cultures. No anterior chamber taps were performed. Seven vitrectomy samples were taken from the six eyes with three intraoperative vitreous samples (43%) producing positive *C. dubliniensis* cultures. The mean length of time for notification of a positive vitreous *C. dubliniensis* culture was 5.4 days (range 3–10 days). In terms of antifungal sensitivities, all six isolates were sensitive to fluconazole, 5-flucytosine, and voriconazole; however, only three isolates were sensitive to amphotericin B.

### Treatment

In terms of initial treatment, five eyes (83%) from four patients were treated with vitreous tap, intravitreal injection of an antifungal agent, and systemic antifungal therapy on the day of presentation. One eye from one patient was initially treated with urgent vitrectomy, intravitreal injection of an antifungal agent, and systemic antifungal therapy on the day of presentation (17%).

Systemically, the empirical antifungal agent administered was fluconazole in two patients (40%), voriconazole in two patients (40%), and amphotericin B in one patient. Once positive *C. dubliniensis* culture was obtained, three patients were treated with systemic fluconazole (60%) and two patients were treated with systemic voriconazole (40%). For the patients treated with fluconazole, the mean duration of treatment was 40 days (range 36–42 days). For the patients treated with voriconazole, the mean duration of treatment of 40.5 days (range 35–46 days).

In terms of ophthalmic treatment, all six infected eyes received intravitreal antifungal injections. A total of 33 intravitreal injections were given - 22 voriconazole and 11 amphotericin B. The mean number of intravitreal injections per eye was 5.5 (range 3–10), and the mean interval between intravitreal injections was 4.2 days.

All eyes underwent vitrectomy at least once for clearance of infection. The mean time from onset of symptoms to the first vitrectomy was 20.5 days (range 1–43 days). Only one vitrectomy was performed urgently on the day of presentation for diagnosis and for early clearance of infection due to extensive vitreous snowballs. In total, eight vitrectomies were performed on the six eyes - two eyes required two vitrectomies due to subsequent retinal detachments which required repair.

### Visual outcomes and mortality

There was no associated mortality in our series, and no infected eye required enucleation or evisceration. No patient developed any secondary fungal infection during follow-up or experienced any systemic complications.

In terms of visual outcomes, five eyes (83%) recovered best-corrected visual acuity of 20/60 or better and one eye had a poor final visual acuity of hand movements only. No eyes developed associated cataract or glaucoma. Two eyes did develop retinal detachment over the course of the endophthalmitis which required surgical repair. Both detachments were caused by single superior retinal tears. The maculae were still attached on the day of diagnosis and repair in both cases. The first of these two patients had initially undergone urgent vitrectomy on the day of presentation, and subsequent retinal detachment occurred 10 days later. This patient eventually had final best-corrected visual acuity of 20/60. The second patient had an initial vitrectomy 12 days after presentation and developed retinal detachment 2 days later. This patient eventually had final best-corrected visual acuity of hand movements only.

## Discussion

It is well documented that *Candida* species are among the most common known fungal pathogens. They can cause a wide range of diseases in humans from superficial mucosal infections to life-threatening disseminated diseases. By far, the most prevalent isolated strain is *C. albicans* which has been implicated in up to 65% of cases of candidemia [7]. Although *C. albicans* has been shown to have low virulence in healthy individuals, candidemia is associated with relatively high rates of morbidity and mortality [8]. Among patients with diagnosed candidemia, reported rates of associated endogenous endophthalmitis range from less than 3% to 44% and mortality rates in these patients have been reported as high as 77% [9]. *Candida* species reported to cause endophthalmitis include *C. albicans*, *C. tropicalis*, *C. parapsilosis*, *C. glabrata*, *C. guilliermondii*, *C. krusei*, and most recently *C. dubliniensis*.

*C. dubliniensis* was first described in 1995 in Dublin, Ireland, among HIV-infected patients with oral candidiasis [2]. It has been found to be only a minor component of the oral flora of humans, and although it primarily causes oral candidiasis in HIV-infected and immunocompromised patients, rare reports of invasive systemic infections in both HIV-positive and HIV-negative patients have been documented [10-12]. This is consistent with large epidemiological studies which report that candidemia caused by *C. dubliniensis* has only rarely been identified and represents around 2% of yeast-positive blood cultures [7]. The rare isolation of *C. dubliniensis* has also likely been due to its close phenotypic similarity to *C. albicans* resulting in often misidentification in laboratory settings. In fact, retrospective studies of *Candida* isolates in fungal stock collections going back to the 1970s have since found many cases of *C. dubliniensis* that were mistakenly identified as *C. albicans*[13-15]. This suggests that *C. dubliniensis* has probably been present in the community for a much longer period than its recent discovery indicates and could also suggest that many cases of *C. dubliniensis* endophthalmitis have been wrongly attributed to *C. albicans* in previous published literature.

Thus, due to its rare nature, only recent identification, and probable previous misidentification, *C. dubliniensis* has only rarely been reported as the causative organism in endophthalmitis. Although the sample size of our series is small, surprisingly, our five cases of *C. dubliniensis* endophthalmitis represent the largest single case series published to date with only three other previously reported cases to our knowledge in the literature (Tables 1 and 2) [4-6]. The significance of these now eight total cases is important because recent studies have shown that *C. dubliniensis* is less pathogenic than *C. albicans* and this may have implications for the diagnosis and treatment of endophthalmitis caused by these separate organisms.

Comparing the five cases in our series with the three previous cases (Tables 1 and 2), it is clear that risk factors for endogenous *C. dubliniensis* endophthalmitis include male gender, intravenous drug use, hepatitis, liver disease, placement of an intravenous catheter, and endocarditis. It is also important to note that only one previous patient has been HIV positive [5]. In terms of presentation, often these cases present as isolated endophthalmitis infections without any other systemic evidence of disseminated disease.

Diagnosis in *C. dubliniensis* endophthalmitis can be difficult because the organism has high false-negative rates on fungal cultures of both vitreous samples and blood cultures. However, the sensitivities of these investigations in our series were improved compared to previous *C. albicans* endophthalmitis series [16]. Microbiologically, although fluconazole-resistant isolates of *C. dubliniensis* have been described due to overexpression of genes encoding multidrug transporter proteins [17], all isolates from the reported cases of *C. dubliniensis* endophthalmitis have been susceptible not only to fluconazole but most other conventional antifungal agents (Table 1).

In terms of treatment and outcomes, vitrectomy, repeated intravitreal injection, and systemic antifungal therapy appear to be efficacious in *C. dubliniensis* endophthalmitis with 87% of infected eyes recovering vision of 20/80 or better. Only retinal detachment appears to be associated with poorer visual outcomes, while surprisingly, early vitrectomy, increased number of intravitreal injections, and delayed presentation all appear to not influence visual outcomes.

Most interestingly, the visual outcomes for endogenous *C. dubliniensis* endophthalmitis appear to be slightly better when compared to a recent study into visual outcomes in endogenous *C. albicans* endophthalmitis cases [16]. In this study, 33% of patients with *C. albicans* endophthalmitis had a final visual acuity of 20/200 or worse and 52% of patients had a final visual acuity of 20/40 or worse [16]. These differences in visual outcomes between *C. dubliniensis* endophthalmitis and the more common *C. albicans* endophthalmitis support the hypothesis suggested by Moran et al. that *C. dubliniensis* is less pathogenic than *C. albicans* due to the decreased ability of *C. dubliniensis* to produce hyphae and its intolerance to environmental stressors [18]. This is further supported by the reduced associated systemic mortality in *C. dubliniensis* endophthalmitis.

## Conclusions

Overall, *C. dubliniensis* is a rare cause of both candidemia and endogenous endophthalmitis and can present in both HIV-positive and HIV-negative patients. Based on the albeit limited number of reported endophthalmitis cases caused by this organism, we recommend treatment with intravitreal voriconazole to avoid possible amphotericin B resistance, followed by vitrectomy for clearance of infection and a 6-week course of systemic fluconazole therapy. Although this organism can be resistant to fluconazole, there is no documented case of *C. dubliniensis* endophthalmitis where the isolate has shown this resistance. In addition, although this treatment regime may be complicated by retinal detachment, overall it appears to be associated with improved visual outcomes compared to cases caused by *C. albicans* and does not lead to any associated systemic morbidity or mortality.

## Competing interests

The authors declare that they have no competing interests.

## Authors’ contributions

JP conceived the study and drafted the manuscript. TM identified the patients, reviewed the medical records, conducted the literature review, and drafted the manuscript. Both authors read and approved the final manuscript.
